# Interdisciplinary Occupational Health System for Recovery From Musculoskeletal Injuries in US Army Personnel

**DOI:** 10.1001/jamanetworkopen.2026.34391

**Published:** 2026-09-17

**Authors:** Timothy A. Murray, Andrew G. Thompson, Axel Wolff, Melissa D. Richardson, Abe R. Dummar, Kevin A. Bigelman

**Affiliations:** 1 US Army Research Institute of Environmental Medicine, Natick, Massachusetts; 2 Holistic Health and Fitness, US Army Maneuver Center of Excellence, Ft. Eustis, Virginia

## Abstract

**Importance:**

Musculoskeletal injuries are a leading cause of pain, disability, and lost workforce productivity in the US. These injuries are more frequent in physically demanding occupations, making strategies to accelerate recovery a critical economic, public health, and workforce sustainability issue.

**Objective:**

To evaluate whether an embedded interdisciplinary occupational health intervention was associated with faster return-to-work times after a musculoskeletal injury.

**Design, Setting, and Participants:**

This cohort study analyzed longitudinal data on 43 870 526 person-month observations from active-duty US Army personnel collected between October 1, 2017, and September 30, 2025.

**Exposure:**

Assignment to an Army unit with an embedded Holistic Health and Fitness (H2F) occupational health system, which includes on-site physical therapists, athletic trainers, occupational therapists, registered dietitians, strength and conditioning coaches, and cognitive performance specialists.

**Main Outcomes and Measures:**

Number of limited duty workdays after a musculoskeletal injury and time to return to full duty. Linear regression, logistic regression, and Kaplan-Meier models with inverse probability of treatment weighting were used to estimate the association between the intervention and recovery outcomes.

**Results:**

Among 1 420 195 musculoskeletal injuries in active-duty soldiers (mean [SD] age, 28.6 [7.64] years; 79% male) that resulted in limited duty restrictions, 19% were in soldiers exposed to the H2F embedded occupational health system. Exposure to H2F was associated with a 5.9% (95% CI, 7.2%-4.6%; *P* < .001) reduction in the duration of limited duty workdays compared with soldiers not exposed to H2F. Reductions in limited duty workdays were greater for lower-body injuries than for upper-body injuries. These reductions corresponded to an estimated 3.3 million to 4.0 million workdays restored during the study period, representing an annual labor productivity value of $93 million to $164 million.

**Conclusions and Relevance:**

In this cohort study of active-duty US Army soldiers, embedding an interdisciplinary occupational health system directly into the workplace was associated with a significantly accelerated recovery time from musculoskeletal injuries. These findings suggest that employer-sponsored occupational health interventions are a scalable strategy to reduce injury-related absenteeism and enhance workforce productivity, particularly in high-risk industries.

## Key Points

**Question:**

Is an embedded occupational health intervention associated with faster return-to-work times after musculoskeletal injuries in a workforce with physically demanding occupations?

**Findings:**

In this cohort study of 1 420 195 musculoskeletal injuries among active-duty US army personnel, exposure to the embedded occupational health system was associated with a 5.9% reduction in limited duty workdays.

**Meaning:**

This study suggests that embedding an interdisciplinary occupational health system directly into the workforce represents a scalable strategy to accelerate injury recovery and reduce absenteeism-related economic losses.

## Teaser

This cohort study of active-duty US Army personnel evaluates whether an embedded interdisciplinary occupational health intervention was associated with faster return-to-work times after a musculoskeletal injury.

## Introduction

Musculoskeletal disorders represent one of the most prevalent and economically consequential health conditions in the US. Approximately half of US adults experience a diagnosable musculoskeletal condition during their lifetime, making these disorders a leading cause of pain, disability, and functional limitation among working-age populations.^1^ Musculoskeletal conditions account for nearly 30% of all workplace injuries involving days away from work and are a primary driver of absenteeism and productivity loss nationwide.^2,3^ From a health system perspective, musculoskeletal disorders generate the highest aggregate health care expenditures of any diagnostic category in the US, exceeding cardiovascular disease, cancer, and mental health disorders.^1,4^

Beyond direct medical costs, the indirect burden of musculoskeletal injury—including lost workdays, reduced labor force participation, and early retirement—magnifies its population-level impact. These injuries disproportionately affect physically demanding occupations, including construction, public safety, and military service.^5,6^ As such, musculoskeletal injury is not solely a clinical concern but a workforce sustainability and economic stability issue with broad societal and public health implications.

Physically demanding professions exhibit particularly elevated musculoskeletal injury rates. High incidence has been documented among military personnel,^6,7,8,9^ law enforcement officers and firefighters,^5,10^ youth and high school athletes,^11,12^ and maritime service members.^13^ These occupations involve sustained load carriage, repetitive movement, environmental stressors, and unpredictable physical demands, all of which increase cumulative tissue stress and injury risk. Inadequate recovery, sleep disruption, suboptimal nutrition, and high psychological stress further compound vulnerability to both acute and chronic musculoskeletal conditions.^7,14^ Collectively, these exposures create structural risk environments that traditional episodic medical models may be poorly positioned to address.

The US military operates a large, closed employer health care system, which provides a uniquely informative setting to evaluate musculoskeletal injury prevention and recovery at scale. The US Army systematically tracks occupational health metrics, including functional performance, days of restricted work (ie, limited duty) status, and overall fitness for duty (ie, deployability), enabling direct linkage between injury burden and workforce capacity. Musculoskeletal injuries are the top medical encounters for active-duty personnel and the leading cause of temporary duty limitations, medical discharge, and long-term disability.^15,16,17,18^ When personnel are placed on limited duty, workforce productivity declines, training pipelines are disrupted, and overall organizational capacity is reduced.^19,20^ This high-resolution surveillance infrastructure creates an opportunity to examine how structural changes in injury management are associated with recovery trajectories at the enterprise level.

Traditional injury management in large organizations often relies on clinic-based, referral-dependent care delivered after injury has progressed to functional limitation. In contrast, contemporary public health approaches emphasize early identification, integrated prevention, and embedded care models designed to shorten recovery time and prevent recurrence. Early access to rehabilitation services has been associated with shorter injury duration and improved functional outcomes in physically active populations.^21,22^ However, enterprise-wide implementation of interdisciplinary, embedded performance health systems remains uncommon outside elite sport settings.^23,24,25^

In response to persistent injury burden and its impact on fitness for duty, the US Army implemented the Holistic Health and Fitness (H2F) system, representing one of the largest enterprise-wide occupational health interventions to date.^26^ The H2F system embeds interdisciplinary teams consisting of 22 specialists within Army brigades to address 5 interrelated domains of occupational health: physical, nutritional, mental, spiritual, and sleep. These teams include physical therapists, athletic trainers, occupational therapists, registered dietitians, cognitive performance specialists, and strength and conditioning coaches. Brigades, the Army’s primary organizational and operational unit (analogous to large corporate divisions), typically consist of 3000 to 5000 service members for training and deployment, representing a ratio of 1 specialist for every 136 to 227 soldiers. The eAppendix in Supplement 1 provides a detailed breakdown of the H2F system and rollout to Army brigades. The H2F system provided flexibility for each brigade to determine the unique processes to use the interdisciplinary teams to meet their occupational needs. The H2F model shifts care from reactive referral-based treatment to proactive, early intervention infrastructure by colocating injury prevention and rehabilitation services within the operational environment. Beginning in 2021, the Army began a rollout of H2F across operational brigades, with a plan of full Army implementation by 2029 and the National Guard and Army Reserves piloting the program starting in 2027.

Although the H2F system was developed within a military context, it is comparable with whole systems approaches used in collegiate and professional athletics^27,28,29^ and represents a scalable occupational health systems model with potential relevance in additional settings. Public safety agencies, industrial labor sectors, and organized athletics similarly experience substantial musculoskeletal injury–related absenteeism and disability. Evaluating the population-level association of an embedded, interdisciplinary, early intervention system model with recovery outcomes provides insight for policymakers and occupational health leaders seeking to reduce injury burden and preserve workforce capacity.

The purpose of this investigation was to evaluate the association of H2F as an embedded occupational health system with return-to-work trajectories after musculoskeletal injury in a large cohort of active-duty military personnel. We hypothesized that implementation of the H2F system would be associated with faster recovery times for return to full work after a musculoskeletal injury. Given the national burden of musculoskeletal disorders and their implications for disability, labor force participation, and health care expenditure, these findings may inform broader public health strategies aimed at workforce sustainability and injury prevention.

## Methods

### Data Source

This cohort study analyzed 43 870 526 person-month observations of active-duty US Army soldiers serving between October 1, 2017, and September 30, 2025, using administrative and medical data obtained from the Soldier Performance, Health, and Readiness Database, which maintains historical personnel and medical records from more than 25 Department of War systems of record and is housed at the US Army Research Institute of Environmental Medicine (USARIEM). The specific systems of record used in this analysis include the Defense Manpower Data Center, Integrated Personnel and Pay System–Army, Medical Operational Data System, and the Digital Training Management System. The primary exposure in this analysis was a soldier being in a brigade resourced with H2F in a given month.^26^ In primary analyses, soldiers who transferred from brigades that have fielded H2F to those that have not were excluded to minimize exposure misclassification. Sensitivity analyses included in the eMethods in Supplement 1 include these individuals to assess potential carryover effects, as well as a restricted analysis only including soldiers from 2021 to 2025 to ensure the COVID-19 pandemic did not influence the results. This study analyzed a limited dataset, which was determined to be exempt from institutional review board review under 45 CFR 46.104(d)(4) by the USARIEM Office of Research Quality and Compliance and were granted a waiver of Health Insurance Portability and Accountability Act authorization from the US Army Medical Research and Development Command’s institutional review board to allow for the granular use of dates, demographic variables, and locations (Protocol #25-13-E). Because this study analyzed preexisting data where no member of the study team had contact with any of the individuals and this study was determined to be exempt from institutional review board review, informed consent was not required for this study. This study followed the Strengthening the Reporting of Observational Studies in Epidemiology (STROBE) reporting guideline for cohort studies.

When a service member sustains an injury requiring activity restriction, a medical professional assigns a temporary work restriction (administratively called a limited duty profile) specifying injury type, injury severity, anatomical location, cause of injury, work restriction, and limited duty duration. Musculoskeletal injuries were identified using profile body system codes. Upper-body injuries included elbow, wrist, hand, upper arm, lower back, shoulder, middle back, neck, and forearm. Lower-body injuries included ankle, foot, thigh, hip, knee, and lower leg. Preventable musculoskeletal injuries are injuries that could be prevented by modified behaviors (eg, changes in running gait), training (eg, development of undertrained muscles), or occupational exposures (eg, changes in work conditions or uniforms) in which H2F could have an influence (eg, running, load carriage, strength training), consistent with established military injury epidemiology classifications.^6,7^ The primary outcome in this analysis is days spent on limited duty from musculoskeletal injuries. Covariates included in these analyses include age, sex, race and ethnicity (American Indian or Alaska Native, Asian, Hispanic or Latino, Native Hawaiian or Pacific Islander, non-Hispanic Black, non-Hispanic White, and unknown or other race or ethnicity [Asian Indian, Chinese, Filipino, Guamanian, Japanese, Korean, Vietnamese, Other Asian Descent, Aleut, Eskimo, US or Canadian Indian Tribes, Melanesian, Micronesian, Polynesian, Other Pacific island descent, other, none, or unknown as defined by the Army administrative system of record]), military rank, and Military Occupational Specialty (MOS; a code used to define a soldier’s specific job in the Army), used to classify soldiers as combat or noncombat, which may contribute to different types and severity of injuries sustained in the course of their job duties. Race and ethnicity were obtained from the US Army authoritative soldier administrative human resources system of record and used to control for social or cultural factors that may influence the outcome. Additional data processing details are provided in the eMethods in Supplement 1. During the study period, 1 420 195 unique musculoskeletal injuries resulted in restricted work duty. Recovery duration analyses were restricted to injured personnel. Models estimating the probability of restricted work included the full active-duty population. Descriptive statistics for both the full sample and the sample who required a work restriction can be found in eTables 1 and 2 in Supplement 1.

### Statistical Analysis

To evaluate the association of the H2F system with recovery from musculoskeletal injuries, we used 3 complementary empirical approaches. First, we estimated a series of linear regressions to compare differences in limited duty days from musculoskeletal injuries between personnel exposed to H2F and those who were never exposed. These models included controls for age, sex, race and ethnicity, rank, combat MOS status, injury severity, an individual fixed effect to control for time-invariant unobserved factors, and a fiscal year fixed effect to control for system-wide shocks (eg, COVID-19–related disruptions). Because the distribution of limited duty days was right skewed, the natural log of limited duty days was used as the dependent variable, for which we report the exponentiated coefficients, which allows for the regression coefficients to be interpreted as a percentage change in the expected number of limited duty days.

Second, we conducted survival analyses using unadjusted Kaplan-Meier curves, which compared recovery trajectories between groups, with differences assessed using the log-rank test. In the survival analysis, there is no censoring, as every soldier would complete their administrative limited duty medical profile before any assessments were made for separation from the Army.

Third, to evaluate population-level exposure, we estimated logistic models estimating the probability of monthly musculoskeletal-related limited duty placement. The logistic regression models included controls for age, sex, race and ethnicity, rank, combat MOS status, and a fiscal year fixed effect. For these models, we report post hoc marginal effects, which show percentage-point differences in the probability of sustaining a musculoskeletal injury resulting in restricted work.

To address nonrandom assignment of H2F, we applied inverse probability of treatment weighting (IPTW) based on observed covariates to all models to ensure that compositional differences between H2F-exposed and H2F-nonexposed personnel do not impact the outcomes.^30^ IPTW is calculated by weighting the non-H2F–resourced observations by *p*(*s*)/[*p* − *p*(*s*)], where *p*(*s*) is the propensity score estimated using logistic regression with age, sex, race and ethnicity, military rank, and an indicator for being in a combat MOS as covariates. Separate propensity scores were estimated for the whole population vs the injured population. Standard errors for all models are clustered at the brigade (Unit Identification Code) level to account for group effects that may bias statistical inference because H2F was not assigned at the individual level.^31,32^ eTables 1 and 2 in Supplement 1 show the impact of IPTW.

To contextualize restored workdays in economic terms, we applied Department of War composite military personnel cost constructs used for workforce valuation to estimate the association of H2F with economic labor value. Prior H2F economic modeling incorporated Defense Finance and Accounting Service composite enlisted compensation rates^33^ to estimate the labor value associated with restricted work status.^34^ Based on standard annual enlisted compensation of $50 000 to $75 000, the daily workforce value is estimated between $140 and $205 per service member.

We conducted all analyses using Stata MP, version 19.0 (StataCorp LLC). For linear regression results, we report exponentiated regression coefficients with 95% CIs. For logistic regression results, we report marginal effects with 95% CIs. In all analyses, we considered 2-sided *P* < .05 to be statistically significant.

## Results

This study evaluated 1 420 195 musculoskeletal injuries among active-duty soldiers (mean [SD] age, 28.6 [7.6] years; 79% male and 21% female; 2% American Indian or Alaska Native, 6% Asian, 17% Hispanic or Latino, 1% Native Hawaiian or Pacific Islander, 28% non-Hispanic Black, 47% non-Hispanic White, and 1% unknown or other race or ethnicity; 21% combat MOS). A total of 19% of the cohort exposed to H2F (eTable 2 in Supplement 1).

### Limited Duty Duration After Musculoskeletal Injury

**Figure 1.**
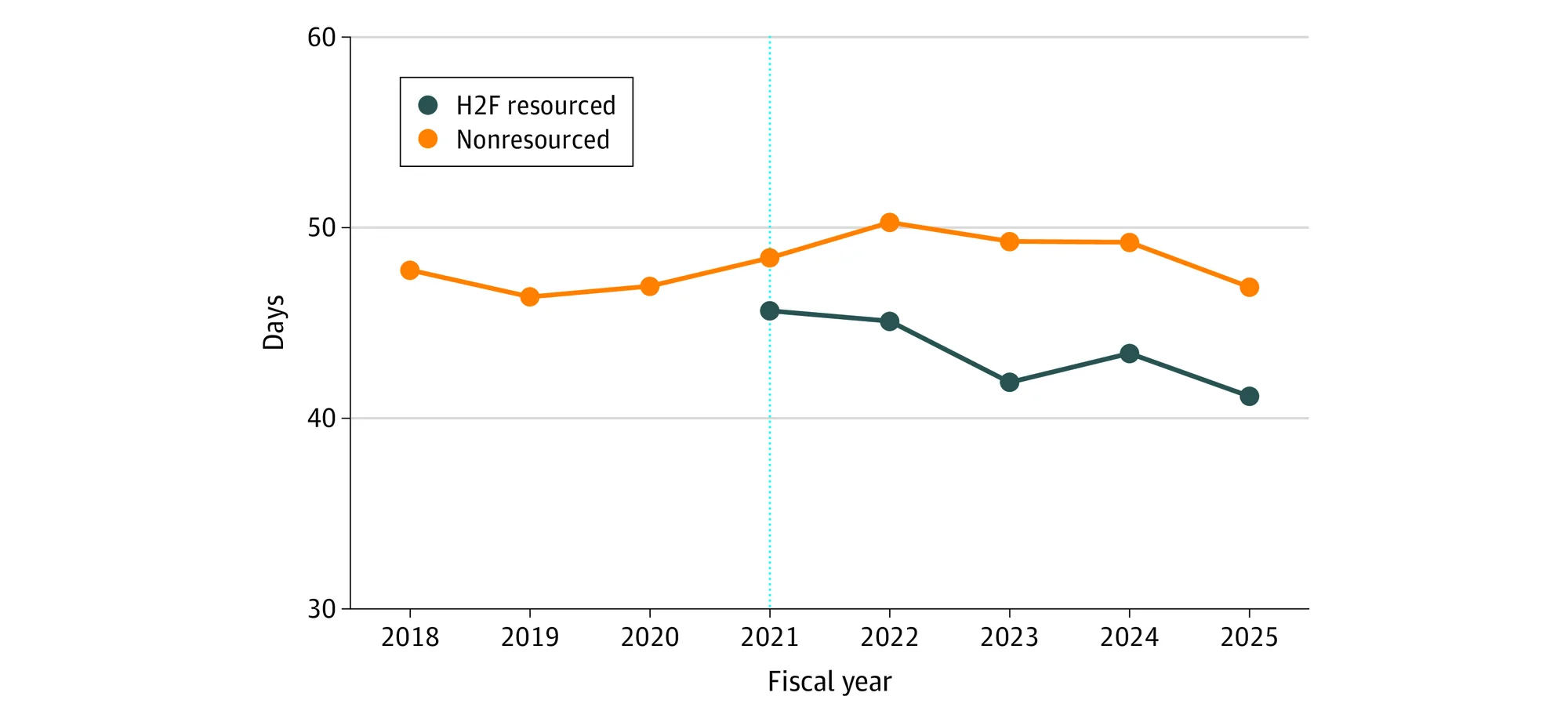
Line Graph of Mean Days Spent on Limited Duty for Musculoskeletal Injury This line graph shows the mean days soldiers spent on a limited duty temporary profile for soldiers who were resourced by the Health and Holistic Fitness System (H2F) and those who were not. The vertical dashed line at 2021 indicates when H2F resourcing began.

Figure 1 displays mean restricted work duration by H2F exposure status. In the first year after implementation, personnel in H2F-exposed brigades had a mean (SD) of 2.7 (0.3) fewer days of work restrictions per musculoskeletal injury compared with personnel in nonexposed brigades. By 2025, this difference increased to 5.7 (0.2) days, indicating progressive divergence in recovery duration over time.

**Figure 2.**
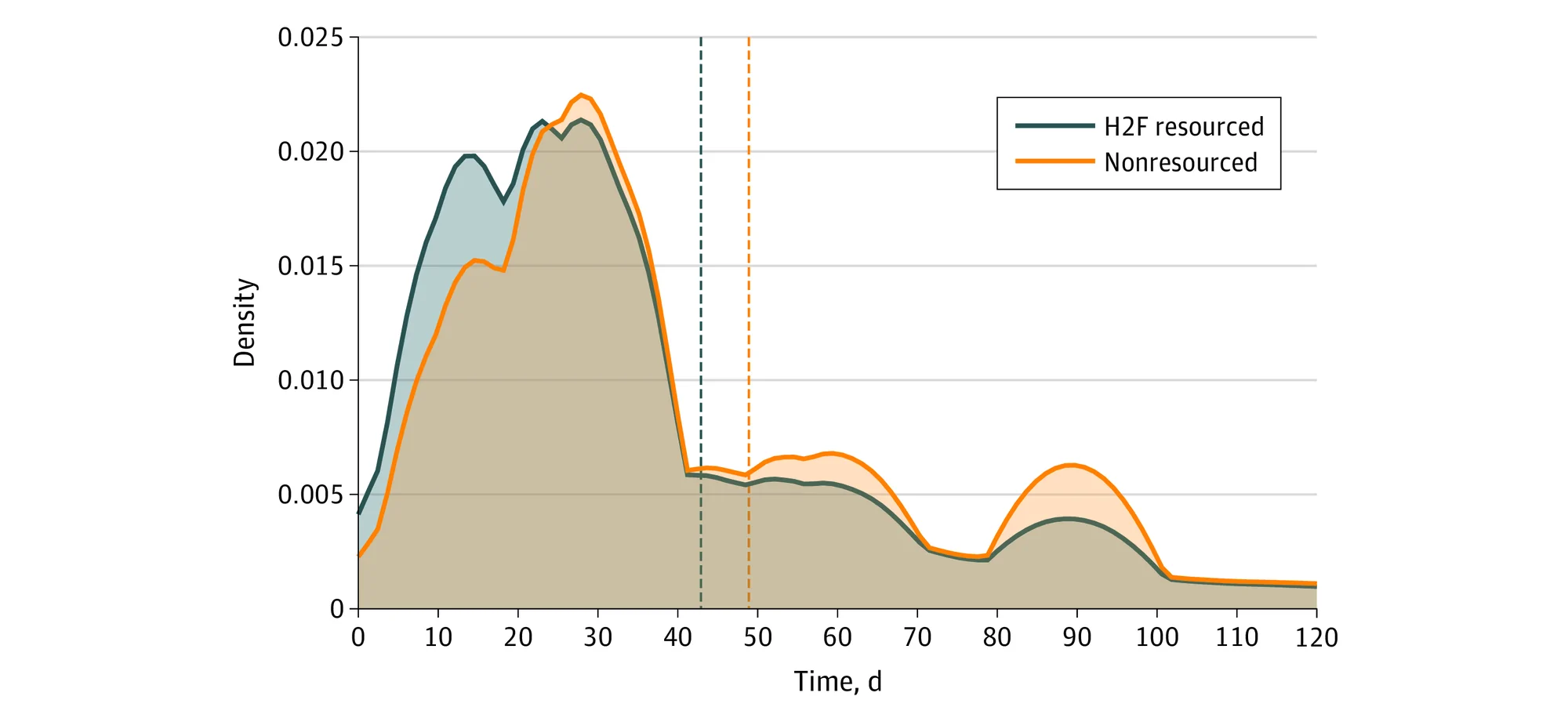
Kerndel Density Plot of Distribution of Days Spent on Limited Duty for Musculoskeletal Injury This kernel density plot shows a bandwidth of 5 for days spent on a limited duty temporary profile for soldiers who were resourced by the Health and Holistic Fitness System (H2F) and those who were not from fiscal year 2021 to fiscal year 2025. Vertical dashed lines indicate the mean values over that period.

Figure 2 presents the distribution of restricted workdays. Most musculoskeletal injuries resulted in fewer than 40 restricted-duty days; however, the entire distribution for H2F-exposed personnel shifted leftward. On average, H2F exposure was associated with approximately 6 fewer restricted work days per injury episode.

**Figure 3.**
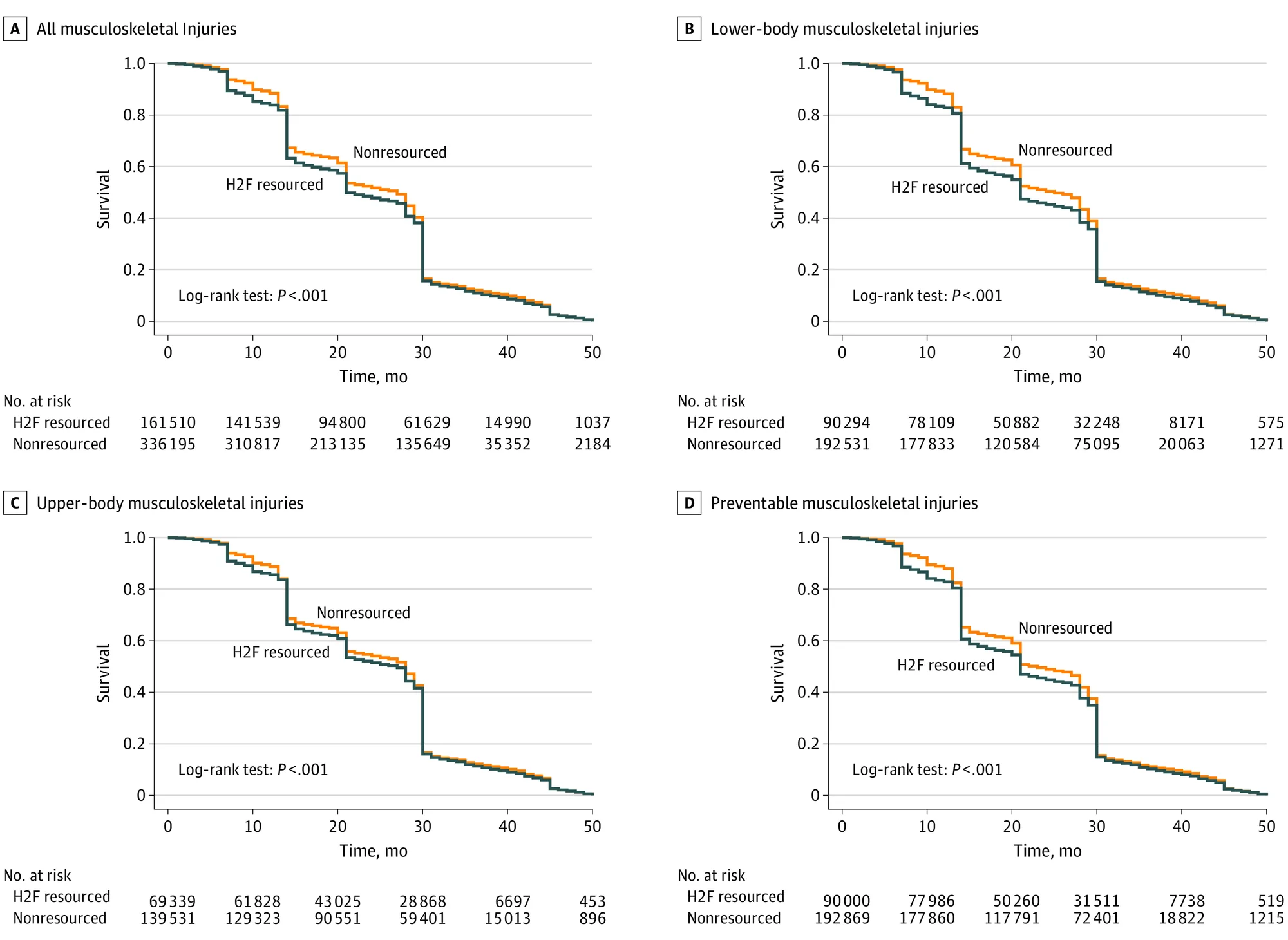
Kaplan-Meier Survival Estimates for Return to Duty After Musculoskeletal Injuries These survival plots show the Kaplan-Meier estimates for survival time after musculoskeletal injuries for the Health and Holistic Fitness System (H2F)-resourced and -nonresourced soldiers. A lower survival time means a faster recovery time and faster return to duty after a musculoskeletal injury.

Kaplan-Meier curves (Figure 3) demonstrate consistently faster return to work among H2F-exposed personnel across all injury categories. Median recovery times were shorter for personnel in H2F-exposed brigades, and log-rank tests indicated statistically significant differences in recovery distributions (χ^2^_1_ = 1475.88; *P* < .001). Linear regression estimates indicate that H2F exposure was associated with a reduction in work restriction duration across all musculoskeletal injuries (coefficient, −5.9 [95% CI −7.2 to −4.6]; *P* < .001) (Table). Reductions were larger for lower-body injuries (coefficient, −6.9 [95% CI, −8.6 to −5.2]; *P* < .001) and moderate for upper-body injuries (coefficient, −3.8 [95% CI, −5.6 to −1.9]; *P* < .001). Injuries classified as potentially preventable were also significantly reduced (coefficient, −5.6 [95% CI, −7.5 to −3.7]; *P* < .001). Across models, estimates suggest approximately 2 to 6 fewer restricted duty days per injury, depending on baseline duration.

**Table.** Regression Results for the Association of H2F With Musculoskeletal Injury Recovery Time

Characteristic	All musculoskeletal injuries		Lower-body injuries		Upper-body injuries		Preventable musculoskeletal injuries	
	Coefficient (95% CI)	*P* value	Coefficient (95% CI)	*P* value	Coefficient (95% CI)	*P* value	Coefficient (95% CI)	*P* value
H2F vs non-H2F^a^	−5.9 (−7.2 to −4.6)	<.001	−6.9 (−8.6 to −5.2)	<.001	−3.8 (−5.6 to −1.9)	<.001	−5.6 (−7.5 to −3.7)	<.001
*R* ^2^	0.416	NA	0.463	NA	0.462	NA	0.459	NA
No.	1 159 449	NA	563 266	NA	439 846	NA	512 383	NA

Abbreviations: H2F, Health and Holistic Fitness System; NA, not applicable.

^a^ Linear regression coefficients are exponentiated so they can be interpreted as relative effects.

### Likelihood of Limited Duty Placement

Logistic regression results indicate that personnel in H2F-exposed brigades were more likely to be placed on work restriction for a musculoskeletal injury in a given month (marginal effect, 0.008 [95% CI, 0.007-0.009]; *P* < .001) (eTable 3 in Supplement 1). Although modest in magnitude, this result is consistent with enhanced surveillance and earlier identification of injury due to embedded access to rehabilitation personnel. This finding occurred concurrently with shorter recovery duration, suggesting earlier intervention rather than increased injury incidence.

### Population-Level Outcomes

Across the study period, 1 150 813 musculoskeletal injuries resulted in work restriction for personnel who were not exposed to H2F. If the reduction of approximately 5 to 6 restricted work days per injury observed in H2F-exposed personnel were applied to the nonexposed population, an estimated 660 000 to 800 000 productive workdays per year would have been restored since the introduction of H2F in 2021. Although this calculation assumes stable treatment effects across injury types and does not adjust for recurrence or severity, it illustrates how modest individual-level improvements can translate to substantial aggregate workforce gains.

### Economic Labor Productivity Outcomes

Applying the daily workforce value of $140 to $250 per day per soldier to the projected 660 000 to 800 000 restored days results in a total annual economic value of approximately $93 000 000 to $164 000 000 in labor productivity. These estimates reflect conservative workforce valuation only and do not account for the cost of administering H2F (eg, labor expense of H2F personnel, facilities and maintenance expenses, supplies), downstream medical cost avoidance, training replacement expenses, disability prevention, or secondary productivity effects. Even under these constrained assumptions, the aggregate value demonstrates how modest reductions in injury duration can be scaled to substantial enterprise-level economic labor value.

### Robustness Checks

For robustness checks, in eTable 4 in Supplement 1, we report the regression results in which we included soldiers who switched from an H2F-resourced brigade to a nonresourced brigade. In eTable 5 in Supplement 1, we report regression results that restrict the sample to only the years in which H2F was active (October 2020 to September 2025). Both of these specifications show similar findings to our main results.

## Discussion

In this large longitudinal evaluation, exposure to H2F, an embedded occupational health system, was associated with shorter duration of work restriction (ie, limited duty) after musculoskeletal injury and faster return to work across injury categories. Reductions in restricted work days ranged from 3.8% to 6.9%, indicating faster recovery among personnel exposed to H2F, with larger reductions for lower-body injuries. The larger reduction observed for lower-body injuries may reflect the high occupational load carriage and ambulatory demands among military populations,^6,14^ although this possibility warrants further biomechanical investigation. In occupational health settings, enhanced embedded access to clinicians can increase early detection while reducing downstream severity and duration of disability.^35^ These results suggest that if the entire Army had been outfitted with H2F, the number of available personnel would have been higher, amounting to millions of restored productive workdays thanks to less time spent on work restriction per injury.

Embedded performance teams can address modifiable determinants of musculoskeletal injury risk, including load management, progressive rehabilitation, nutritional adequacy, sleep optimization, and environmental risk mitigation.^7,14,36,37,38^ In addition, colocation of these teams within organizational units (ie, brigades) may also reduce barriers to evaluation and shorten time to treatment, mechanisms previously associated with improved recovery duration.^21,22^ Furthermore, personnel having daily access to the occupational health teams can improve trust with the system,^39^ and higher trust is associated with patients being less likely to delay medical care.^40^ This suggests that a possible mechanism underlying shorter recovery duration is reduced time to evaluation and earlier initiation of rehabilitation. In centralized referral-based systems, delays between injury onset and rehabilitation are associated with prolonged disability and increased health care utilization.^35,41^ Personnel having direct access to the interdisciplinary occupational health teams reduces administrative barriers, appointment delays, and fragmented care pathways compared with the traditional model of having to schedule an appointment at a military treatment facility or civilian clinic.

Evidence across civilian and military populations indicates that earlier access to physical therapy is associated with reduced imaging utilization, lower opioid exposure, improved function, and shorter recovery duration.^21,42^ Morgan et al^22^ demonstrated similar associations among physically active populations, and Whitehurst et al^43^ reported substantially shorter time to physical therapy evaluation among H2F-resourced brigades. Although this study was not designed to formally test mediation pathways, the convergence of accelerated evaluation and shorter restricted work duration supports a plausible early intervention mechanism.

Beyond access alone, interdisciplinary integration may contribute additional benefits. Coordinated load modification, progressive rehabilitation, conditioning maintenance, and risk factor modification (eg, sleep, nutrition) align with multidisciplinary models that improve musculoskeletal outcomes compared with fragmented care.^35^

In 2022, the US Army overhauled its annual physical fitness testing to move from the Army Physical Fitness Test to the Army Combat Fitness Test. The Army Combat Fitness Test incorporated strength and power-based exercises, including a 3-repetition maximum deadlift, a standing power throw, and a sprint-drag-carry. However, Hicks et al^44^ found that the switch to a new physical fitness test did not change the pattern of observed injuries.

### Workforce and Public Health Implications

Even modest reductions in injury duration can yield substantial aggregate workforce gains in large occupational systems. Applying a conservative 5% reduction in restricted work days across non-H2F units suggests that hundreds of thousands of productive workdays annually could be restored Army-wide. At a national level, musculoskeletal disorders remain among the leading contributors to disability and work limitation,^1^ and occupational musculoskeletal injuries account for a significant proportion of lost workdays across industries.^3^

These findings suggest that structural early intervention models, particularly those embedding rehabilitation expertise within operational environments, may represent an underused workforce sustainability strategy. High-demand sectors such as public safety, construction, transportation, and organized athletics face similar injury burdens.^5^ Although contextual differences exist between military and civilian systems, the underlying mechanism of earlier access reducing duration of disability is consistent with occupational health literature.^35^

Traditional musculoskeletal management models in large organizations often rely on episodic, referral-based care. Such structures may unintentionally prolong time to evaluation and increase administrative friction. Policies that support embedded, interdisciplinary early intervention models may reduce cumulative lost workdays in high-risk sectors. In sectors with concentrated physical demands, structural integration of early musculoskeletal triage and rehabilitation may mitigate prolonged absenteeism and downstream disability.

Although the colocation of these teams provides several possible benefits, it also comes with potential risks of increased low-value care that could create unnecessary testing and diagnoses to meet patient expectations and, in a civilian setting, fear of medical malpractice litigation, which could, in turn, increase out-of-pocket medical expenses to patients and/or increase administrative costs to the employer.^40,45^ The design and implementation of interdisciplinary occupational health systems require due diligence to balance the benefits of such an initiative while not drastically increasing medical costs.

### Limitations

Several limitations warrant consideration. First, the H2F rollout was not randomized. Although we used IPTW, individual fixed effects, and clustered standard errors to strengthen causal inference, unmeasured confounding cannot be fully excluded. Second, exposure was defined at the unit (ie, brigade) level and did not capture individual-level H2F utilization intensity. Variation in engagement with performance teams may attenuate observed effect sizes. Third, limited duty profiles represent an administrative proxy for recovery duration and do not capture symptom severity, recurrence, long-term functional outcomes, or whether the injury occurred in the unit in which it was reported or in a previous unit. Fourth, the economic translation is illustrative and based on composite compensation constructs rather than comprehensive cost accounting. Estimates exclude downstream medical savings, recurrence reduction, training replacement costs, and disability prevention and therefore likely represent conservative lower-bound approximations. Fifth, system of record data does not include comprehensive physiological and biological data that are risk factors in injury and recovery. Sixth, findings derive from a centralized military health system with standardized occupational demands. Although mechanistic insights may generalize to other physically demanding sectors, effect magnitudes may differ in decentralized civilian contexts.

## Conclusions

In this retrospective cohort study of active-duty US Army soldiers evaluating more than 43 million person-month observations, exposure to an embedded, interdisciplinary occupational health system (H2F) was associated with shorter recovery duration after musculoskeletal injury and accelerated return to work. Individual-level reductions were modest but translated into millions of restored productive workdays at the enterprise level, with conservative economic labor productivity estimates between $93 000 000 and $164 000 000 annually. These findings provide strong evidence that structural integration of early rehabilitation access within occupational settings is a high-value and scalable strategy to mitigate musculoskeletal-related workforce disruption. As musculoskeletal disorders continue to impose substantial economic and productivity burdens nationally,^1^ embedded performance health systems warrant consideration as population-level interventions capable of preserving workforce capacity while improving functional recovery trajectories.

## Notes

Supplement 1. **eAppendix.** US Army Holistic Health and Fitness (H2F) System Overview **eMethods.** Soldier Medical Profiles **eTable 1.** Descriptive Statistics for the Full Sample **eTable 2.** Descriptive Statistics for the Restricted Sample of Soldiers Who Were Placed on Limited Duty **eTable 3.** Logistic Regression Results for Likelihood of Musculoskeletal Injury Resulting in Limited Duty **eTable 4.** Regression Results for the Impact of H2F on Musculoskeletal Injury Recovery Time (Including Switchers) **eTable 5.** Regression Results for the Impact of H2F on Musculoskeletal Injury Recovery Time (All Soldiers Restricted to Fiscal Years 2021-2025)
eReference.

Supplement 2.
Data Sharing Statement
