# Supplementary material for: Interdisciplinary Occupational Health System for Recovery From Musculoskeletal Injuries in US Army Personnel
Source: JAMA Netw Open. 2026 Sep 17;9(9):e2634391. doi: 10.1001/jamanetworkopen.2026.34391 (PMC13586896; doi:10.1001/jamanetworkopen.2026.34391)
Supplement: Supplementary file 1 [file jamanetwopen-e2634391-s001.pdf]

Supplementary Online Content

Murray TA, Thompson AG, Wolff A, Richardson MD, Dummar AR, Bigelman KA. Interdisciplinary occupational health system for recovery from musculoskeletal injuries in US Army personnel. *JAMA Netw Open.* 2026;9(9):e2634391. doi:10.1001/jamanetworkopen.2026.34391

eAppendix. US Army Holistic Health and Fitness (H2F) System Overview

eMethods. Soldier Medical Profiles

eTable 1. Descriptive Statistics for the Full Sample

eTable 2. Descriptive Statistics for the Restricted Sample of Soldiers Who Were Placed on Limited Duty

eTable 3. Logistic Regression Results for Likelihood of MSK Injury Resulting in Limited Duty

eTable 4. Regression Results for the Impact of H2F on MSK Injury Recovery Time (Including Switchers)

eTable 5. Regression Results for the Impact of H2F on MSK Injury Recovery Time (All Soldiers Restricted to Fiscal Years 2021-2025)

eReference.

This supplementary material has been provided by the authors to give readers additional information about their work.

© 2026 Murray TA et al. *JAMA Network Open.*

eAppendix. US Army Holistic Health and Fitness (H2F) System Overview

Field Manual (FM) 7-22, Holistic Health and Fitness, is the official doctrine that outlines the U.S. Army's H2F system. H2F represents a significant cultural and doctrinal shift to an individualized, data-driven approach aimed at optimizing Soldier performance, reducing injury rates, and enhancing the ability of Soldiers to execute their specific job functions (i.e., military readiness).

The H2F system establishes clear policies, regulations, and quality control measures to ensure consistent and effective implementation across the Army. The H2F system is organized around five key domains:

1. Physical Readiness: Develops strength, power, speed, and endurance required for combat tasks, moving beyond simple aerobic fitness.
2. Nutritional Readiness: Emphasizes performance-based nutrition strategies.
3. Mental Readiness: Focuses on building cognitive and interpersonal skills to enhance resilience, decision-making, and the ability to adapt and thrive under stress.
4. Spiritual Readiness: Develops the personal qualities needed to endure hardship and tragedy by drawing on an individual's core values, beliefs, and sense of purpose, regardless of their religious or philosophical worldview.
5. Sleep Readiness: Establishes strategies to optimize sleep duration, timing, and continuity to optimize brain health, cognitive function, and physical recovery.

Each Army brigade that is resourced with H2F receives an H2F Performance Team (HPT) that is comprised of the following personnel:

- 1 Program Director: Advises the brigade command team on performance readiness and integrated H2F into the mission planning and personnel decisions.
- 2 Physical Therapists: Expert in evaluating, diagnosing, and treating musculoskeletal injuries.
- 1 Physical Therapist Specialist: Works with Army Physical Therapist to rehabilitate Soldiers recovering from injury and illness to reduce pain and promote recovery.
- 4 Athletic Trainers: Work alongside the Physical Therapists to provide immediate preventative care and acute injury management in the training environment.
- 2 Registered Dietitians: Translate clinical evidence into practical nutrition plans through individual counselling and group education.

© 2026 Murray TA et al. *JAMA Network Open.*

- 1 Nutrition Care Specialist: Works with Army Registered Dietitian to implement foundational performance nutrition education and promote a positive nutrition environment.
- 2 Occupational Therapists: Focuses on improving cognitive performance, mental, emotional, and interpersonal skills through 1:1 and group training.
- 1 Occupational Therapist Assistant: Works with Army Occupational Therapist to help Soldiers with cognitive, emotional and interpersonal skills through tailored training plans.
- 1 Cognitive Performance Specialist: Manages and provides cognitive enhancement education in individual and group settings.
- 7 Strength and Conditioning Coaches: Develop periodized individualized physical training programs based around Soldiers' individual and unit tasks.

In addition to the HPTs, H2F resourced brigades are also supported by standardized Soldier Performance Readiness Centers (SPRCs), which serve as a centralized location for physical training, injury management, and education.

H2F Rollout to the U.S. Army

Beginning in fiscal year 2021 (October 2020), the Army began a phased rollout of H2F to brigades. Details of that rollout are below:

**Fiscal Year**	**Brigade Types**	**Count**
2021	Brigade Combat Teams	11
	Sustainment Brigades	3
	Aviation Brigades	4
	Field Artillery Brigades	2
	Medical Brigades	2
	Military Police Brigades	2
	Engineering Brigades	2
	Air Defense Artillery Brigades	2
	TOTAL	*28*
2023	Brigade Combat Teams	5
	Sustainment Brigades	1
	Aviation Brigades	1
	Training Brigades	5
	TOTAL	*12*
2024	Brigade Combat Teams	2
	Sustainment Brigades	1
	Aviation Brigades	1
	Special Forces Groups	2
	Psychological Operations Groups	2
	Training Brigades	1

© 2026 Murray TA et al. *JAMA Network Open.*

	Civil Affairs Brigades	1
	TOTAL	*10*
2025	Brigade Combat Teams	9
	Sustainment Brigades	2
	Aviation Brigades	2
	Field Artillery Brigades	1
	Medical Brigades	1
	Training Brigades	3
	Calvary Regiments	3
	TOTAL	*21*

© 2026 Murray TA et al. *JAMA Network Open.*

eMethods. Soldier Medical Profiles

This section outlines how data on Soldier medical "profiles" were processed to be used in data analyses and not double count injuries.

In the U.S Army, when a Soldier is injured, they are placed on a limited duty "profile" by a medical provider. The "profile" defines whether the condition is temporary or permanent, duty restrictions, the body system of the injury (i.e., musculoskeletal, cardiology, behavioral health, dental, etc.), a focus area (e.g., lower extremity, spine, upper extremity, etc.), location detail (e.g., knee, ankle, wrist, foot, etc.), cause of the injury (e.g., slip/trip/fall, running, weight lifting, work task, sports, etc.), start date, and expiration date. In this study, we only included temporary profiles with musculoskeletal system conditions.

These "profiles" can be modified during follow up visits with medical providers, so it is common for a Soldier has multiple records for the same injury. Specifically, the expiration date may be extended to allow for additional time for the injury to recover. When the "profiles" are updated, the start date may change and may occur after the initial expiration date, depending on the date the Soldier was able to see a medical provider. For this study, we evaluate active-duty Soldiers who were on a temporary "profile" for musculoskeletal injuries. We assumed that any "profile" of injury with the same focus area and location were the same injury as long as the expiration date of the first profile and the start date of the subsequent profile were within 10 days of each other. Where there were multiple records for the same injury, we kept only one record and used the first start date and last expiration date to calculate the number of days the "profile" lasted to ensure we did not double count injuries. If a Soldier has multiple temporary musculoskeletal "profiles" that started in the same month, we only used the "profile" with the longest duration.

© 2026 Murray TA et al. *JAMA Network Open.*

eTable 1. Descriptive Statistics for the Full Sample

	All	Raw Unweighted Means		IPTW Weighted Means	
		Non-Resourced	H2F Resourced	Non-Resourced	H2F Resourced
H2F Resourced	0.17	0.00	1.00	0.00	1.00
	(0.38)	(0.00)	(0.00)	(0.00)	(0.00)
Age	29.31	29.54	28.20	27.46	28.20
	(7.89)	(8.12)	(6.57)	(6.74)	(6.57)
Male	0.84	0.84	0.87	0.87	0.87
	(0.36)	(0.37)	(0.34)	(0.34)	(0.34)
Combat MOS	0.25	0.23	0.32	0.31	0.32
	(0.43)	(0.42)	(0.47)	(0.46)	(0.47)
White, Non Hispanic	0.54	0.54	0.53	0.54	0.53
	(0.50)	(0.50)	(0.50)	(0.50)	(0.50)
Black, Non-Hispanic	0.22	0.22	0.22	0.21	0.22
	(0.41)	(0.41)	(0.41)	(0.41)	(0.41)
Asian	0.06	0.06	0.06	0.05	0.06
	(0.23)	(0.23)	(0.24)	(0.22)	(0.24)
Native Hawaiian or Pacific Islander	0.01	0.01	0.01	0.01	0.01
	(0.09)	(0.10)	(0.07)	(0.10)	(0.07)
American Indian or Alaska Native	0.01	0.01	0.02	0.01	0.02
	(0.12)	(0.12)	(0.14)	(0.12)	(0.14)
Unknown or Other Race	0.01	0.01	0.01	0.01	0.01

© 2026 Murray TA et al. *JAMA Network Open.*

	(0.10)	(0.10)	(0.09)	(0.09)	(0.09)
Hispanic or Latino	0.15	0.15	0.16	0.16	0.16
	(0.36)	(0.36)	(0.37)	(0.37)	(0.37)
Observations	43,870,528	36,266,828	7,603,700	36,266,350	7,603,700

Note: standard deviations in parentheses.

© 2026 Murray TA et al. *JAMA Network Open.*

eTable 2. Descriptive Statistics for the Restricted Sample of Soldiers Who Were Placed on Limited Duty

	All	Raw Unweighted Means		IPTW Weighted Means	
		Non-Resourced	H2F Resourced	Non-Resourced	H2F Resourced
H2F Resourced	0.19	0.00	1.00	0.00	1.00
	(0.39)	(0.00)	(0.00)	(0.00)	(0.00)
Age	28.55	28.85	27.27	27.06	27.27
	(7.64)	(7.88)	(6.34)	(6.52)	(6.34)
Male	0.79	0.78	0.82	0.81	0.82
	(0.41)	(0.42)	(0.38)	(0.39)	(0.38)
Combat MOS	0.21	0.19	0.29	0.26	0.29
	(0.40)	(0.39)	(0.45)	(0.44)	(0.45)
White, Non Hispanic	0.47	0.47	0.46	0.46	0.46
	(0.50)	(0.50)	(0.50)	(0.50)	(0.50)
Black, Non-Hispanic	0.28	0.28	0.27	0.28	0.27
	(0.45)	(0.45)	(0.45)	(0.45)	(0.45)
Asian	0.06	0.06	0.06	0.06	0.06
	(0.23)	(0.23)	(0.24)	(0.23)	(0.24)
Native Hawaiian or Pacific Islander	0.01	0.01	0.01	0.01	0.01
	(0.09)	(0.09)	(0.07)	(0.09)	(0.07)
American Indian or Alaska Native	0.02	0.02	0.02	0.02	0.02
	(0.13)	(0.13)	(0.15)	(0.13)	(0.15)
Unknown or Other Race	0.01	0.01	0.01	0.01	0.01

© 2026 Murray TA et al. *JAMA Network Open.*

	(0.09)	(0.10)	(0.07)	(0.07)	(0.07)
Hispanic or Latino	0.17	0.16	0.18	0.17	0.18
	(0.37)	(0.37)	(0.38)	(0.38)	(0.38)
Observations	1,420,278	1,150,792	269,486	1,150,692	269,486

Notes: Standard deviations in parentheses.

© 2026 Murray TA et al. *JAMA Network Open.*

eTable 3. Logistic Regression Results for Likelihood of MSK Injury Resulting in Limited Duty

	(1) All[a]		(2) No Switchers[b]	
	Coefficient [95% CI]	P-Value	Coefficient [95% CI]	P-Value
Log Odds	0.150 [0.129,0.172]	<0.001	0.233 [0.197,0.256]	<0.001
Marginal Effect[c]	0.005 [0.004,0.006]	<0.001	0.008 [0.007,0.009]	<0.001
N	43,867,543		35,401,064	

[a] Column 1 shows the results of the logistic regression showing the effect of H2F on the likelihood of receiving an MSK injury that resulted in limited duty for all Soldiers.

[b] Column 2 restricts the results to exclude any Soldier who switched from a H2F resourced brigade to a non-resourced brigade. The log odds are the logistic regression coefficient.

[c] The post hoc marginal effect shows the difference in the predicted probability of sustaining an MSK injury that resulted in limited duty. of H2F on all MSK injuries.

Abbreviations: H2F = Holistic Health and Fitness. MSK = Musculoskeletal.

© 2026 Murray TA et al. *JAMA Network Open.*

eTable 4. Regression Results for the Impact of H2F on MSK Injury Recovery Time (Including Switchers)

	(1) All MSK Injuries		(2) Lower Body		(3) Upper Body		(4) Preventable MSK Injuries	
	Coefficient [95% CI]	P-Value	Coefficient [95% CI]	P-Value	Coefficient [95% CI]	P-Value	Coefficient [95% CI]	P-Value
H2F vs Non-H2F[a]	-5.786 [-7.026, -4.546]	<0.001	-6.607 [-8.206, -5.008]	<0.001	-3.864 [-5.556, -2.173]	<0.001	-5.623 [-7.425, -3.820]	<0.001
R^2	0.407		0.456		0.456		0.452	
N	1,225,271		596,335		467,064		543,438	

[a] Linear regression coefficients are exponentiated so they can be interpreted as relative effects. Abbreviations: H2F = Health and Holistic Fitness System. MSK = Musculoskeletal.

© 2026 Murray TA et al. *JAMA Network Open.*

eTable 5. Regression Results for the Impact of H2F on MSK Injury Recovery Time (All Soldiers Restricted to Fiscal Years 2021-2025)

	(1) All MSK Injuries		(2) Lower Body		(3) Upper Body		(4) Preventable MSK Injuries	
	Coefficient [95% CI]	P-Value	Coefficient [95% CI]	P-Value	Coefficient [95% CI]	P-Value	Coefficient [95% CI]	P-Value
H2F vs Non-H2F[a]	-5.117 [-6.801, -3.433]	<0.001	-5.515 [-7.668, -3.363]	<0.001	-2.460 [-4.693, -0.227]	0.031	-4.579 [-7.086, -2.071]	<0.001
R^2	0.438		0.481		0.475		0.482	
N	727,193		345,126		279,132		308,986	

[a] Linear regression coefficients are exponentiated so they can be interpreted as relative effects. Abbreviations: H2F = Health and Holistic Fitness System. MSK = Musculoskeletal.

© 2026 Murray TA et al. *JAMA Network Open.*

eReference.

Department of the Army. FM 7-22 Holistic Health and Fitness. 2020. https://armypubs.army.mil/epubs/DR_pubs/DR_a/ARN44522-FM_7-22-002-WEB-7.pdf
