# Supplementary material for: Interdisciplinary Occupational Health System for Recovery From Musculoskeletal Injuries in US Army Personnel
Source: JAMA Netw Open. 2026 Sep 17;9(9):e2634391. doi: 10.1001/jamanetworkopen.2026.34391 (PMC13586896; doi:10.1001/jamanetworkopen.2026.34391)
Supplement: Supplementary file 2 [file jamanetwopen-e2634391-s002.pdf]

Data Sharing Statement

Murray. Interdisciplinary Occupational Health System for Recovery From Musculoskeletal Injuries in US Army Personnel. *JAMA Netw Open*. Published September 17, 2026.
doi:10.1001/jamanetworkopen.2026.34391

Data
Data available: No

Additional Information
Explanation for why data not available: The data used in this study come from proprietary Department of Defense systems of record and were used under strict data sharing agreements and are not publicly available.
